# Primary Failure to an Anti-TNF Agent in Inflammatory Bowel Disease: Switch (to a Second Anti-TNF Agent) or Swap (for Another Mechanism of Action)?

**DOI:** 10.3390/jcm10225318

**Published:** 2021-11-15

**Authors:** Javier P. Gisbert, María Chaparro

**Affiliations:** Gastroenterology Unit, Hospital Universitario de La Princesa, Instituto de Investigación Sanitaria Princesa (IIS-IP), Universidad Autónoma de Madrid (UAM), Centro de Investigación Biomédica en Red de Enfermedades Hepáticas y Digestivas (CIBEREHD), 28006 Madrid, Spain; mariachs2005@gmail.com

**Keywords:** anti-TNF, inflammatory bowel disease, primary failure, ustekinumab, vedolizumab

## Abstract

Background: About a third of patients with inflammatory bowel disease do not respond to anti-tumour necrosis factor (anti-TNF) therapy, which is challenging. Aim: To review the current data on the two main strategies when facing primary non-response to an anti-TNF agent in inflammatory bowel disease: changing to a second anti-TNF (switching) or to a drug with another mechanisms of action (swapping). Methods: We performed a bibliographic search to identify studies reporting on efficacy of any biologic treatment after primary anti-TNF non-response. Results: The efficacy of a second anti-TNF is lower when the reason to withdraw the first one is primary failure. Nevertheless, switching to another anti-TNF even after primary failure may still be effective in some patients. Both vedolizumab and ustekinumab have generally been shown to be less effective in anti-TNF exposed patients. However, despite primary anti-TNF failure, patients may respond to vedolizumab or ustekinumab in a limited but considerable number of cases. The cause for swapping (primary vs. secondary anti-TNF failure) seems to have limited effect on vedolizumab efficacy. Primary anti-TNF non-response seems to be a clearer predictor of treatment failure for ustekinumab. Unfortunately, the two main strategies to treat specifically a patient with primary non-response to an anti-TNF agent—switching to a second anti-TNF or swapping for vedolizumab/ustekinumab—have not been properly compared. Conclusion: The data reviewed in the present study clearly emphasise the imperative need to carry out head-to-head randomised trials in patients exposed to anti-TNF agents in general, and specifically in those with primary non-response to these agents.

## 1. Introduction

Inflammatory bowel diseases (IBD)—ulcerative colitis (UC) and Crohn’s disease (CD)—are chronic idiopathic inflammatory diseases affecting the gastrointestinal tract. Anti-tumour necrosis factor (anti-TNF) agents have revolutionised the treatment of IBD. Their use avoids the need for steroid therapy, promotes mucosal healing, reduces hospitalisations and surgeries, and therefore, dramatically improves the quality of life of IBD patients [1].

However, approximately one-third of patients do not respond to anti-TNF induction therapy (known as primary non-responders) [2,3,4]. In addition, a subset of patients who initially respond to anti-TNF drugs discontinue therapy because they lose their response (secondary non-responders) or develop an intolerable adverse event [5]. Finally, the restricted time period of the therapy in some countries affects the clinical course of the disease and entails the need to resume biological therapy [6].

In patients who lose their response to an anti-TNF drug, it is well established that a switch to another anti-TNF agent in an attempt to regain remission is an accepted option [7,8]. To date, however, few studies have evaluated the effectiveness of a second anti-TNF agent in IBD patients who did not achieve remission (that is, in primary failures) with their first anti-TNF drug [9].

Some studies have shown that previous anti-TNF therapy is associated with heightened probability of primary treatment failure of subsequent anti-TNF therapy [2]. Patients with primary non-response may have altered pharmacokinetics (rapid drug clearance resulting in low trough levels) or pharmacodynamics (mechanistic failure due to non-TNF-mediated inflammation), resulting in decreased likelihood of response to a second anti-TNF agent.

For many years, anti-TNF agents were the only type of biologics available for IBD treatment. However, new drugs that target different inflammatory pathways have been approved for IBD in recent years: two biologics (vedolizumab [10] and ustekinumab [11]), and one small molecule (tofacitinib) [12]. As the armamentarium for IBD increases in coming years, it will become important to understand factors associated with response in order to best position and personalise therapy [13].

The efficacy of vedolizumab and ustekinumab in IBD has been studied in randomised placebo-controlled trials. Almost half of the patients included in these Phase 3 registration trials were anti-TNF naïve [14,15], while observational cohort studies have shown that most patients receiving vedolizumab and ustekinumab in daily practice are anti-TNF exposed [16]; and the same holds true for tofacitinib [17]. At present, there is no clear indication on the most appropriate second-line therapy in case of failure to an anti-TNF agent and, in the absence of head-to-head comparative efficacy data, the choice between a second anti-TNF agent or the other alternatives is often based on clinician’s personal experience, drug availability, and economic issues. It is generally considered that patients who have failed treatment with anti-TNF agents are a more refractory group, but this seems to be valid not only for second-line anti-TNF therapy but also for any other biologic agent.

In summary, although both treatment options—switching to a second anti-TNF or swapping for a drug with another mechanism of action—may be effective for achieving and maintaining remission in IBD patients failing an anti-TNF treatment, it is still unclear how these two strategies should be positioned specifically after primary anti-TNF failure. With the increasing number of treatments approved for IBD management, positioning of drugs becomes a pertinent question. In fact, both switching and swapping strategies have been considered suitable approaches for other immune-mediated diseases [18].

The present review will summarise the current data on the two main strategies to treat a patient with primary non-response to an anti-TNF agent: changing to a second anti-TNF (switching) or to a drug with another mechanism of action such as vedolizumab, ustekinumab or tofacitinib (swapping).

## 2. Bibliographic Search

A bibliographic search was designed to identify studies reporting on efficacy of any of these regimens with biologics or small molecules after primary non-response to an anti-TNF agent. An electronic search was performed in PubMed up to April 2021 using the following algorithm: (“inflammatory bowel disease” OR “ulcerative colitis” OR Crohn) AND (anti-TNF OR antiTNF OR infliximab OR adalimumab OR certolizumab OR golimumab) AND (vedolizumab OR ustekinumab OR tofacitinib). In addition, a complementary electronic search with the following algorithm was used: (“inflammatory bowel disease” OR “ulcerative colitis” OR Crohn) AND (anti-TNF OR antiTNF OR infliximab OR adalimumab OR certolizumab OR golimumab) AND (vedolizumab OR ustekinumab OR tofacitinib) AND (metaanalysis OR meta-analysis OR “systematic review”). In addition, the reference lists from the selected articles were reviewed to identify additional studies of potential interest. Only studies conducted in humans were included, whereas animal models were excluded. Available data from both clinical trials and “real life” studies were included. Pharmacokinetic studies (e.g., therapeutic drug monitoring) were excluded. Studies evaluating the response in patients with post-operative recurrence of CD were also excluded. Articles published in any language were included.

## 3. Switch to Another Anti-TNF Agent

### 3.1. Rationale for Switching to a Second Anti-TNF Agent in Primary Anti-TNF Failures

The mechanism underlying primary non-response to an anti-TNF drug and subsequent response to another anti-TNF drug is unknown. In view of the similar structure and function shared by infliximab and adalimumab (or any other anti-TNF agent), one might predict that patients who do not have a primary response to one of these agents will not respond to the other. Thus, the lack of efficacy of the anti-TNF agent in these patients could be due to specific disease characteristics in which TNF does not play a pivotal role (i.e., it is not a major pro-inflammatory cytokine responsible for the inflammation), suggesting that other pathways could be implicated in the pathogenesis of the disease. In fact, in a patient with primary non-response to one medication, the routine clinical practice is to move out of that mechanism of action.

However, in some studies, a considerable proportion of primary non-responders to infliximab had a sustained clinical benefit with adalimumab (or vice versa), indicating that the primary failure to a biologic anti-TNF agent does not necessarily translate across the class of therapy. It has been suggested that differences in efficacy between anti-TNF agents may be due to differences in pharmacokinetic properties or tissue penetration [19].

### 3.2. Efficacy of a Second Anti-TNF Agent Depending on the Cause for Switching: Primary vs. Secondary Failure

Only a few studies with small sample size have analysed the effectiveness of the sequential use of a second anti-TNF if the reason for withdrawal of the first drug was primary failure. The rates of remission in these studies were highly variable, ranging from 0% to more than 50%. Studies evaluating the efficacy of a second anti-TNF agent after primary failure with a first one, are summarised in Table 1 [20,21,22,23,24,25,26,27,28,29,30,31,32,33,34,35,36,37,38,39]. The mean efficacy (remission rate) in this scenario (primary failure, 20 studies, with a total of 410 patients) was 41%.

The efficacy of a second anti-TNF agent after secondary—instead of primary—failure with a first one, considering these same studies (to avoid possible biases as much as possible), is also summarised in Table 1. Criteria used for primary failure definition in these studies are included in Table S1. The mean efficacy (remission rate) in this scenario (secondary failure) was 49%, that is, slightly higher than in patients with primary failure. This difference in efficacy was confirmed when only studies providing information both after primary and secondary failure were considered: 36% vs. 49%, respectively.

The aforementioned results are in agreement with a previous meta-analysis, which concluded that the efficacy of a second anti-TNF in CD patients largely depends on the cause for switching: the remission rate was higher when the reason to withdraw the first anti-TNF was intolerance (61%), compared with secondary (45%) or primary failure (30%) [8].

In summary, the efficacy of a second anti-TNF depends on the cause for switching, being lower when the reason to withdraw the first anti-TNF is primary failure.

### 3.3. Studies Evaluating the Efficacy of Switching to a Second Anti-TNF Agent in Primary Failures to a First One

Two large studies, the CHOICE trial [31] and the CARE trial [32], evaluated clinical effectiveness in a cohort of patients with CD, including a subgroup of patients with primary failure to infliximab. These trials demonstrated the efficacy of a second anti-TNF after primary failure with the first agent, although neither was specifically designed to assess the response of an anti-TNF drug in this scenario.

The first study specifically designed to evaluate the effectiveness of a second anti-TNF agent after primary failure of the first one (including CD patients who did not achieve remission with the first agent) was recently published [34]. The first anti-TNF was discontinued because of (complete) non-response in 54% of patients and partial response in 46%. As much as 51% of patients achieved remission in the short-term with the second anti-TNF agent. A trend towards differences in remission rates between patients with a partial response and complete non-response to a previous anti-TNF drug was reported (59% vs. 44%), suggesting that perhaps the former could be better candidates to receive a second anti-TNF agent. Accordingly, the relatively good results described above could be explained by the inclusion of partial responders among patients considered to have experienced primary failure. Finally, a significant proportion of patients lost the response to the second anti-TNF in the long term, although increasing the dose was an effective strategy for regaining response in some cases. Dose escalation in patients who lose the response to anti-TNF drugs is effective and common in clinical practice [40,41]. However, there are insufficient data to recommend dose optimisation strategies in patients who do not respond to the second anti-TNF drug and experienced primary failure with the first one.

The largest study to date (including 1122 IBD patients) in which the strategy of switching to a second anti-TNF after intolerance, primary failure, or secondary failure to the first one has been evaluated, has recently been published [21]. Primary failure (22% of the cases) was considered when the patient did not achieve remission after having received the induction doses of the anti-TNF (including non-responders and partial responders). Unexpectedly, the authors did not find any difference between patients who switched due to primary failure or secondary failure (39% vs. 42%). However, patients who switched to a second anti-TNF due to intolerance to the first drug, had higher remission rates than those who switched due to primary failure (52% vs. 39%).

In summary, some patients (although not many) may still benefit from switching to another anti-TNF agent even if the reason to withdraw the first anti-TNF was primary non-response, suggesting that primary failure to anti-TNF therapy may not necessarily be a class-effect phenomenon.

### 3.4. Switch from a Subcutaneous to an Intravenous Anti-TNF Agent

Probably due to historical reasons (infliximab was the first anti-TNF to be commercialised), the experience with the switch from a subcutaneous (sc) agent to infliximab is more limited. It has been hypothesised that intravenous (iv), weight-based TNF dosing could offer advantages over sc, fixed-dose TNFs and may be effective despite primary non-response to previous sc fixed-dose TNFs. Viola et al. investigated IBD outcome after a switch from sc to the iv agents, and showed that patients who switched due to loss of response did numerically, but not statistically, better than patients who switched due to primary failure [39]. In another study, patients with CD and primary non-response to one or more sc TNFs who subsequently received the iv anti-TNF (infliximab) were evaluated, showing clinical remission in as much as 73% of the cases [23]. It is well known that, in some cases, primary non-response may be due to inadequate biologic drug levels [42]. Taking into account that infliximab is the only weight-based iv anti-TNF agent, and that pharmacokinetics can be affected by patient characteristics, such as the body mass index [43], some authors hypothesise that a weight-based regimen may be effective even in those patients with primary non-response to a fixed-dose sc TNF therapy, and therefore a change to a different class of biologic therapy may not be required [39]. In summary, it is possible that prior classification of primary non-response to sc TNF therapy actually represents, at least in some cases, failure to reach adequate drug levels rather than mechanistic failure of TNF therapy.

### 3.5. Third Anti-TNF Therapy

Interestingly, it has been shown that several patients who experienced primary failure with a second anti-TNF finally responded to a third one. This suggests that these agents (infliximab, adalimumab, certolizumab and golimumab), which have different structural, pharmacokinetic and pharmacological properties, may also differ in their modes of action [44]. A recent systematic review showed that only two retrospective studies, with a small sample size and limited follow up, have evaluated the effectiveness of a third anti-TNF in patients whose two previous anti-TNFs had failed [45]. A favourable (albeit limited) efficacy of this—third-line—switching strategy was reported: in the study by Allez et al. [46], clinical response was observed in 51% of patients at Week 20; in particular, 62% (10/16) of the patients receiving third-line anti-TNF treatment because of primary non-response to the second one finally responded. Subgroup analyses demonstrated similar benefits with certolizumab and adalimumab used as a third-line anti-TNF, suggesting that the order of administration of the latter two drugs does not impact upon the efficacy of the third agent. On the other hand, in the study by de Silva et al. [47], over 50% of patients remained on the third anti-TNF at one year; prior primary non-responders to the first anti-TNF agent were significantly less likely to be able to maintain a third anti-TNF agent [47]. However, unexpectedly, primary non-response to the second anti-TNF agent was not predictive of response to the third anti-TNF. More recently, Casanova et al. [21] reported the largest cohort of patients who switched to a third anti-TNF after intolerance to or failure of the second one. Approximately half of the patients who switched to a third anti-TNF received certolizumab and did so due to primary failure. In the short term, of the 71 patients who were included, approximately 50% achieved remission, whereas two-thirds of them were in remission at 24 months. The incidence of loss of response was 22% per patient-year, which is relatively high. Anyway, the delicate balance between pros and cons means the use of a third anti-TNF after failure of two previous agents should be considered only in patients with no other therapeutic option, and decisions should be taken on an individual basis [45].

### 3.6. Fourth Anti-TNF Therapy

One step further, a small case series suggests that even a fourth anti-TNF drug may have some clinical potential in refractory CD patients with prior triple anti-TNF agent failure [46]. Russi et al. reported a case series of eight difficult-to-treat patients with severe and refractory CD receiving golimumab as an off-label rescue medication and fourth-line anti-TNF agent [48]. All eight patients had previously been treated with all three other TNF antagonists approved for CD (infliximab, adalimumab, and certolizumab) without durable clinical response. Five (63%) patients responded after induction, and three (37%) had a continuous clinical response under golimumab. Nevertheless, it should be noted that all patients included in this study were previously anti-TNF responders to at least one agent, and that primary anti-TNF non-responders were not considered for off-label golimumab treatment.

### 3.7. Anti-TNF Reintroduction

Finally, the benefit of anti-TNF (infliximab) reintroduction after successive failure of infliximab and adalimumab has recently been assessed [49]. Patients with CD who received and discontinued successively infliximab and adalimumab, and who were re-exposed to infliximab, were identified. Remission was achieved in 42% at Weeks 6–8 after infliximab re-induction. Although information on the effectiveness was not provided separately for primary and secondary failures, it is mentioned that the results were similar. Thus, the authors concluded that, for CD patients who successively failed infliximab and adalimumab, reintroducing infliximab is feasible and often clinically efficient.

## 4. Swap for Vedolizumab

### 4.1. Efficacy of Vedolizumab in Anti-TNF Naïve vs. Anti-TNF Exposed Patients

Some studies in IBD patients have shown that previous anti-TNF therapy is a risk factor for failure of treatment with vedolizumab [13,14,50,51,52,53,54,55,56], regardless of the reason for anti-TNF discontinuation; conversely others have shown that the response to vedolizumab is independent of previous anti-TNF failure [57,58,59,60,61,62,63]. Nevertheless, it should be noted that, to date, most of the patients who received vedolizumab treatment, both in randomised controlled trials and in clinical practice, were anti-TNF experienced [64].

Patients with anti-TNF-failure represent a highly treatment-experienced population with clinical features that could explain, at least in part, the lower/slower inductive efficacy. For example, compared with TNF-naïve patients, those with prior TNF antagonist treatment failure had, in most of the studies, longer duration of disease and more complicated disease, including prior surgery and histories of fistulas and extraintestinal manifestations.

Two post hoc analyses from the GEMINI studies reported efficacy of vedolizumab in UC and CD patients based on prior anti-TNF history [53,54]. In CD patients, post hoc analyses of the efficacy data for 516 TNF-naïve patients and 960 patients with TNF-failure (including primary non-response, loss of response, or intolerance) from the GEMINI 2 and GEMINI 3 trials were evaluated [53]. Rate of response and remission were numerically higher in CD patients receiving vedolizumab as a first biologic agent than in patients who had experienced TNF failure (48% and 27% vs. 40% and 22% at Week 10). These higher rates persisted at Week 52. The same results were found in UC patients, where patients naïve to anti-TNF had higher rates of response than patients with anti-TNF failure compared to placebo at Week 6 (absolute differences of 15% and 7%, respectively), but the absolute difference in remission rates observed was the same in the maintenance therapy [54].

Furthermore, the response to vedolizumab seems to be faster in the anti-TNF-naïve population [65]. In UC, vedolizumab patients had better symptom scores (rectal bleeding of 0 and stool frequency score of 0 or 1) than placebo at Week 2 (19% vs. 10%), Week 4 (28% vs. 15%), and Week 6 (34% vs. 17%). In CD, only the TNF antagonist-naïve population had better symptom scores (stool frequency and abdominal pain) at Week 0 and Week 2 [65]. Finally, the advantage of vedolizumab in anti-TNF naïve vs. exposed patients has been confirmed when considering endoscopic outcomes [56].

Recently, a clinical prediction model and tool for vedolizumab therapy in CD was developed with GEMINI 2 participants and then validated in the VICTORY cohort. One of the predictors included in this tool was the absence of prior exposure to anti-TNF. This model suggested that the ideal positioning of vedolizumab in CD patients should be prior to anti-TNF exposure (i.e., used as a first-line biologic agent). However, these results are similar for anti-TNF users, where response rates are also better in patients naïve to biologic agents [66]. Finally, some real-practice vedolizumab studies have demonstrated that, in the medium-long term, anti-TNF exposed patients are almost twice more likely to lose response to vedolizumab than anti-TNF naïve patients [67].

In summary, vedolizumab has generally been shown to be more effective in anti-TNF-naïve than in anti-TNF exposed patients.

### 4.2. Efficacy of Vedolizumab in Primary Failures to Anti-TNF Treatment

The information from the pivotal studies on the efficacy of vedolizumab, specifically in patients with primary anti-TNF failure, is quite limited, and is summarised in Table 2 [14,26,50,53,54,68]. Thus, the response rate (note that it is not the remission rate), both in the induction and in the maintenance phase, ranged from 40% to 50%, both in CD and UC patients [69].

In summary, despite primary non-response to an anti-TNF drug, IBD patients may respond to vedolizumab in a considerable number of cases.

### 4.3. Efficacy of Vedolizumab According to the Cause for Swapping: Primary vs. Secondary Anti-TNF Failure

A recent meta-analysis showed no difference in treatment response to vedolizumab for both anti-TNF primary and secondary non-responders [69]. The risk of failing to achieve remission among patients with prior primary non-response to anti-TNF therapy (as compared with patients with prior loss of response to anti-TNF treatment) was observed only in patients treated with ustekinumab (see next section). These results were stable when stratified according to disease type (CD or UC). For example, in the GEMINI trials, vedolizumab showed similar efficacy irrespective of the type of prior TNF antagonist failure—whether inadequate response, loss of response, or intolerance [53]. This equivalence has also been confirmed in a real-world experience, where no difference in outcomes was observed between vedolizumab treated patients who had a primary and secondary loss of response to anti-TNF agents [67]. However, other researchers have reported that the main factor associated with remission at Week 14 of vedolizumab treatment was discontinuation of the first anti-TNF due to loss of response or intolerance as compared to primary non-response [70].

In summary, the cause for swapping (primary vs. secondary anti-TNF failure) seems to have limited effect on the efficacy of vedolizumab treatment. However, a lower efficacy of vedolizumab in patients with primary anti-TNF failure cannot be ruled out.

### 4.4. Comparison between Anti-TNF and Vedolizumab Treatment in Anti-TNF Experienced Patients

Several studies have compared anti-TNF and vedolizumab in anti-TNF experienced patients, although most of them do not provide specific data regarding the type of failure of the anti-TNF treatment (primary failure, loss of response or intolerance). These studies are summarised below.

The first study directly comparing an anti-TNF drug and vedolizumab in a head-to-head approach, which was not a randomised trial, was performed by Allamneni et al. in 2018 [71] and showed similar effectiveness of infliximab and vedolizumab in UC patients when given to anti-TNF experienced patients (including those who did not respond, lost response, or were intolerant), in a real-world setting. Among anti-TNF experienced patients, there was a higher response rate for vedolizumab vs. infliximab (4.52 vs. 2.29 per 100 persons-weeks), but the incidence rate ratio did not reach statistical significance probably due to small sample size.

Mevius et al. [62] showed that vedolizumab therapy was associated with a higher persistence than anti-TNF therapy in IBD patients. However, more patients were bio-experienced in the vedolizumab group (72%) than in the anti-TNF group (24%). Thus, a sub-analysis considering this variable had to be performed: the percentage of patients persistent with index therapy after 12 months was still higher in bio-experienced patients for vedolizumab (71%) than for anti-TNF (55%). However, despite this result the significant higher persistence with vedolizumab, which was mainly driven by the bio-experienced subsample, should be interpreted carefully (in this study and in others). It might be associated with vedolizumab itself, but other potential reasons might also play a role here. Vedolizumab was the only agent in its class at the time, whereas physicians might have easily switched to another anti-TNF agent in case of safety/efficacy concerns regarding an ongoing anti-TNF treatment. Therefore, patients might have stayed longer on vedolizumab therapy in TNF-experienced patients because, in this bio-experienced patient subsample, discontinuing vedolizumab implied changing to another anti-TNF, which would then have been the third anti-TNF agent.

Macaluso et al. [72] compared the effectiveness of the three biologics (adalimumab, golimumab and vedolizumab) in consecutive patients with UC. Overall, half of the patients were exposed to anti-TNF agents, but this percentage was higher in the vedolizumab group. Thus, a propensity score-adjusted analysis was performed. At 12 weeks, similar steroid-free remission rates were reported for the three groups. At 52 weeks, a steroid-free remission was reported in 51% of the patients in the vedolizumab group, in 31% in the adalimumab group, and in 29% in the golimumab group (*p* = 0.002 for vedolizumab vs. adalimumab, *p* = 0.001 for vedolizumab vs. golimumab). Cox survival analysis demonstrated that patients treated with vedolizumab had reduced probability of treatment discontinuation compared to those treated with adalimumab and golimumab. 

In the VARSITY study, a Phase 3, double-blind, randomised, trial conducted at 245 centres in 34 countries, vedolizumab and adalimumab were compared in patients with UC [73]. The general conclusion was that vedolizumab was superior to adalimumab with respect to achievement of clinical remission and endoscopic improvement. Unfortunately, this study was not primarily focused on patients who had failed anti-TNF treatment, and most of the recruited patients were anti-TNF naïve. Among the patients who had previous exposure to an anti-TNF agent, clinical remission rates at Week 52 were numerically greater (but the difference did not reach statistical significance) in both groups: 20% and 16% in the vedolizumab and adalimumab groups, respectively. Previous anti-TNF therapy presented primary failure in 50% of the patients in both groups, but unfortunately no information on the response according to the type of failure—primary or secondary—was provided [74]. Thus, although it could have been hypothesized that adalimumab would be disadvantaged relative to vedolizumab for patients who previously received treatment with an anti-TNF agent, the findings of this study did not suggest this. Nevertheless, the lack of dose escalation in either treatment group (dose escalation is typically performed more frequently with adalimumab than with vedolizumab in clinical practice), may have skewed the results in favour of vedolizumab [75]. Regardless, the VARSITY trial sets the stage for further head-to-head trials to determine the most appropriate treatment in UC, ideally in populations with refractory disease, such as patients in whom TNF inhibitors have failed [76].

Macaluso et al. [77] performed a multicentre, real-life comparison of the effectiveness of vedolizumab and adalimumab in CD. Nearly half of the patients were experienced to biologics. Of note, the differences between both groups were relevant at baseline, as patients treated with vedolizumab had worse predictive factors than those treated with adalimumab (they were less frequently naïve to biologics: 31% vs. 81%). Thus, Cox survival analysis weighted for propensity score was performed, showing no significant difference in the probability of achieving clinical and endoscopic response between the two drugs; in addition, the safety profile was also similar.

Helwig et al. [78] evaluated more than 5000 patients who had received prescriptions for second-line biologic drugs in Germany between 2014 and 2017. Vedolizumab users were matched to adalimumab, golimumab, and infliximab users based on age, sex, and index year. Treatment persistence was higher for vedolizumab than for TNF inhibitors up to three years after initiating second-line biologic therapy. One of the possible hypotheses that could explain the lower risk of treatment discontinuation in patients receiving vedolizumab than in those receiving TNF inhibitors in this study, is the lower frequency of side effects in vedolizumab patients.

Hupé et al. [70] retrospectively compared the efficacy of infliximab and vedolizumab in UC patients from 12 French centres who failed a first sc anti-TNF agent. The main reason for adalimumab/golimumab discontinuation was primary non-response (in 72% of the cases), although unfortunately results were not provided separately for primary and secondary failures. Clinical remission at Week 14 was achieved in 26% of patients treated with infliximab and in a higher proportion (49%) of those treated with vedolizumab. As important baseline differences existed between the two cohorts (for example, patients treated with infliximab had a more severe disease and were more often primary non-responders to the first anti-TNF than patients treated with vedolizumab), a propensity score matching analysis was performed, and this difference remained statistically significant. Survival rates without treatment discontinuation at years one and three were also higher (80% and 55% with vedolizumab, and 50% and 29% with infliximab).

Rundquist et al. [79] compared the effectiveness of anti-TNF agents and vedolizumab in IBD patients previously exposed to a first-line anti-TNF treatment. A propensity score-matched cohort was created using Swedish nationwide registers. For CD, drug survival was similar (73% in the vedolizumab group vs. 74% in the anti-TNF group). For UC, respective figures were also similar: 69% and 62%. The authors could not adjust the analyses by primary non-response and secondary loss of response since this information was not available in the national registers.

Bohm et al. [80] retrospectively compared the effectiveness and safety of vedolizumab and anti-TNF therapy in CD patients, performing a propensity score-weighted comparison. The authors included 1266 patients (659 treated with vedolizumab). Adverse event rates were significantly lower with vedolizumab vs. TNF-antagonist therapy. Notably, however, exploratory subgroup analyses suggested that infliximab might be superior to vedolizumab for the achievement of clinical remission and steroid-free clinical remission in anti-TNF exposed patients. Furthermore, anti-TNF therapy was associated with higher treatment persistence compared with vedolizumab.

In summary, most of the studies that have compared anti-TNF and vedolizumab treatment in anti-TNF experienced patients (without considering the type of failure to the anti-TNF treatment), have suggested better results with vedolizumab than with a second anti-TNF drug. However, this advantage of vedolizumab could not be confirmed in a few studies.

### 4.5. Comparison between Anti-TNF and Vedolizumab Treatment in Primary Failures to Anti-TNF Treatment

Studies comparing second-line anti-TNF agents and vedolizumab specifically in patients with primary failure to anti-TNF treatment are exceptional and are summarised below.

Papamichael et al. [81] studied the long-term outcome of patients with UC and primary non-response to infliximab. The probability for primary non-response was higher in patients who switched to another anti-TNF agent compared with those swapping for vedolizumab. In addition, regarding patients who continued on biologics after primary non-response to infliximab, there was a marginally higher cumulative probability for relapse in patients switching to another anti-TNF agent compared with those swapping for vedolizumab.

More recently, Favale et al. [26] retrospectively compared the efficacy of adalimumab and vedolizumab in UC patients who failed infliximab, from eight Italian IBD referral centres. The reason for infliximab discontinuation differed between the two groups (e.g., primary failures were more frequent in the vedolizumab group than in the adalimumab group, 12% vs. 5%). Overall, at Week 52, 62% and 71% of patients on adalimumab and vedolizumab, respectively, had therapeutic success (non-statistically significant differences). The success rate was significantly higher in the vedolizumab group than in the adalimumab group among infliximab secondary failures. However, no difference in the failure and biologic discontinuation-free survival was observed in the infliximab primary failure subgroup, although the sample size was very small: two out of three (67%) patients in the adalimumab group, and six out of 12 (50%) in the vedolizumab group failed at Week 52. Therefore, the authors concluded that vedolizumab might be the therapy of choice in those UC patients who show secondary failure to infliximab. Unfortunately, no conclusions could be drawn for infliximab primary failures due to the limited number of subjects enrolled.

In summary, studies comparing second-line anti-TNF agents and vedolizumab specifically in patients with primary failure to anti-TNF treatment are exceptional, and the number of patients included is very low. Therefore, no conclusion can be drawn about the best strategy (switch or swap) in the specific scenario of anti-TNF primary non-responders.

## 5. Swap for Ustekinumab

### 5.1. Efficacy of Ustekinumab in Anti-TNF Naïve vs. Anti-TNF Exposed Patients

Some studies, including Phase 3 clinical trials (UNITI trials), have shown that previous anti-TNF therapy is a risk factor for treatment failure with ustekinumab [15,82] whereas others have reported that the response to ustekinumab is independent of previous anti-TNF failure [83,84]. In a recent pooled analysis of real-world evidence, most patients (97.7%) were anti-TNF experienced [16]. Thus, the data available in the literature do not allow an adequate comparison of the effectiveness of ustekinumab between anti-TNF naïve and experienced patients [64,85].

In summary, ustekinumab, as any other biologic, probably achieves a higher efficacy in anti-TNF-naïve than in anti-TNF exposed patients. However, as the experience of ustekinumab in anti-TNF-naïve patients is still quite limited, this conclusion should be interpreted with caution.

### 5.2. Efficacy of Ustekinumab in Primary Failures to Anti-TNF Treatment

The information from the pivotal studies on the efficacy of ustekinumab, specifically in patients with primary anti-TNF failure, is quite limited, and is summarised in Table 3 [15,86]. Thus, the response rate (note that it is not the remission rate), both in the induction and in the maintenance phase, ranged from 40% to 50%, both in CD and UC patients [69].

Schmitt et al. [87] characterised molecular mechanisms that were associated with endoscopic resistance to anti-TNF therapy. Cytokine profiles, cell surface markers, signalling proteins and cell apoptosis were assessed, and it was concluded that expansion of apoptosis-resistant intestinal TNFR2+IL23R+ T cells was associated with resistance to anti-TNF therapy in CD. Thus, IL-23 seems to be centrally involved in mediating resistance to anti-TNF therapy in patients with CD and thereby represents a suitable molecular target in anti-TNF refractory disease.

In summary, as it was the case with vedolizumab, despite primary non-response to an anti-TNF drug, IBD patients may respond to ustekinumab in a limited but considerable number of cases.

### 5.3. Efficacy of Ustekinumab According to the Cause for Swapping: Primary vs. Secondary Anti-TNF Failure

In a recent meta-analysis, treatment response to a second biologic, particularly with ustekinumab, was shown to be poorer in anti-TNF therapy primary non-responders, in comparison with secondary non-responders [69].

In another meta-analysis, where only two randomised controlled trials (CERTIFI and UNITI-1) were included, clinical response was significantly higher for patients who received ustekinumab compared with those who received placebo in secondary non-responders, but in the case of primary non-responders this benefit could not be confirmed [88]. The results of this study suggest that although ustekinumab seems to be an appropriate therapeutic approach in patients with CD who fail to respond to anti-TNF therapy, the best clinical effect can be observed in secondary non-responders to previous anti-TNF treatment, while patients with primary non-response show no significant benefit in terms of clinical response. As an example, in UNITI-1, six-week response was found to be greater in patients with secondary anti-TNF non-response (37%) than primary non-response (24%) [15]. However, the association found between primary anti-TNF failure and prediction of ustekinumab response in this study needs to be interpreted with caution given the limited number of patients who were considered to have history of primary non-response.

More recently, Singh et al. [69] analysed whether response to a second-line biologic varied depending on the reason for discontinuation of the primary anti-TNF agent (primary non-response, secondary loss of response, or intolerance), through a systematic review and meta-analysis. Two randomised controlled trials—CERTIFI and UNITI—were included in the case of ustekinumab. Overall (considering all types of biologics), as compared with patients who discontinued prior anti-TNF due to intolerance, patients with prior primary non-response were 24% less likely to achieve remission with second-line biologics. Of note, the risk failing to achieve remission among patients with prior primary non-response to anti-TNF (as compared with patients with prior loss of response) was observed only in patients treated with ustekinumab; however, as previously stated in the vedolizumab section, there was no difference in response to vedolizumab in patients with prior primary non-response or loss of response to anti-TNF agents in this meta-analysis. These results were maintained when stratified according to disease type (CD or UC).

In summary, anti-TNF primary non-response seems to be a predictor of ustekinumab failure, unlike loss of response.

### 5.4. Comparison between Anti-TNF and Ustekinumab Treatment in Anti-TNF Experienced Patients

Studies comparing second-line anti-TNF agents and ustekinumab specifically in patients with primary failure to anti-TNF treatment are lacking. However, several studies have compared anti-TNF and ustekinumab treatment in anti-TNF experienced patients without considering the type of failure to the anti-TNF treatment (primary failure, loss of response or intolerance). These studies are summarised below.

Ahmed et al. [89] provided a real-world comparison of adalimumab (most cases were TNF-naïve) and ustekinumab (most cases were TNF-experienced) in CD patients. Among TNF-experienced patients, adalimumab was numerically inferior in inducing clinical response (2/8 (25%) vs. 29/56 (52%)) and remission (2/8 (25%) vs. 15/56 (27%)), but neither of these differences were statistically significant.

More recently, Cerpa Arencibia et al. [90] evaluated patients with CD who were on second-line biologic treatment (ustekinumab or anti-TNF) after failure of a first anti-TNF treatment (although the reason for discontinuation was not provided), reporting higher remission rates with ustekinumab (76%) than with anti-TNF treatment (43%).

In summary, studies comparing second-line anti-TNF agents and ustekinumab specifically in patients with primary failure to anti-TNF treatment are lacking. The very few studies that have compared anti-TNF and ustekinumab treatment in anti-TNF experienced patients in general (without considering the type of anti-TNF failure) suggest better results with ustekinumab than with a second anti-TNF drug.

### 5.5. Comparison between Vedolizumab and Ustekinumab Treatment in Anti-TNF Experienced Patients

Randomised controlled trials directly comparing vedolizumab and ustekinumab are currently not available. Most studies indirectly comparing these two drugs in anti-TNF experienced patients do not provide effectiveness results according to the type of failure to the anti-TNF treatment (primary failure, loss of response or intolerance). These studies are summarised below.

Biemans et al. [91] compared vedolizumab and ustekinumab in CD patients who failed anti-TNF treatment (although the reason for discontinuation was not provided) in a prospective registry specifically developed for comparative studies with correction for confounders. All patients had previously received at least one anti-TNF, and 70–80% of the patients had received at least two. To adjust for confounding factors, propensity score matching was performed. Ustekinumab-treated patients were more likely to achieve corticosteroid-free clinical remission (odds ratio, 2.58; 95% confidence interval, 1.36–4.90), biochemical remission, and combined corticosteroid-free clinical and biochemical remission, while safety outcomes were comparable.

Alric et al. [92] compared the effectiveness and safety of ustekinumab and vedolizumab in patients with CD refractory (including both primary and secondary failures) or intolerant to anti-TNF in a retrospective observational cohort. More patients had been exposed to two anti-TNFs in the vedolizumab cohort, but this difference was abolished after propensity weighting. At Week 48, ustekinumab was associated with a higher clinical remission rate (54% vs. 38%) and treatment persistence (71% vs. 50%) than vedolizumab. However, the rate of steroid-free clinical remission did not differ significantly between ustekinumab and vedolizumab.

Townsend et al. [93] compared the effectiveness of ustekinumab and vedolizumab in anti-TNF refractory CD, performing a propensity score matched analysis. Steroid-free remission was higher among ustekinumab-treated patients at two and 12 months. More patients treated with ustekinumab remained on therapy at the end of 12 months. However, a very small number of patients with anti-TNF primary failure (eight in the vedolizumab group, and six in the ustekinumab group) were included. Furthermore, even though the authors conducted sensitivity analyses considering patients who had discontinued therapy for primary non-response, unfortunately results were not provided.

Manlay et al. [94] compared the short- and long-term effectiveness of vedolizumab and ustekinumab in CD patients with prior anti-TNF exposure. After propensity score analysis (ensuring that prior primary anti-TNF non-response was similar for both groups), ustekinumab was more effective than vedolizumab to achieve corticosteroid-free clinical remission at Week 54 (49% vs. 41%) and deep remission at Week 14 (26% vs. 3.8%).

Finally, several meta-analyses have compared the efficacy of different biologic agents for the treatment of IBD. Their general results, and their data in patients with anti-TNF failure, are summarised in Table 4 [69,95,96,97,98,99,100,101,102,103,104,105,106,107]. Those meta-analyses including relevant information for the comparison between vedolizumab and ustekinumab in anti-TNF exposed patients are summarised below.

Pagnini et al. [99] analysed all available evidence regarding efficacy of a second biologic in anti-TNF experienced CD patients, including randomised clinical trials. Indirect comparisons performed with network meta-analysis suggested no specific agent is clearly superior to others. However, primary non-responders treated with anti-TNF after failure were not included. In this same line, Shim et al. [64], when reviewing the role of vedolizumab and ustekinumab for the treatment of IBD, performed indirect comparisons by separate groups and did not observe any significant difference between these two drugs in achieving clinical remission in anti-TNF-experienced CD patients.

Kawalec et al. [98] performed an indirect comparison of ustekinumab vs. vedolizumab in patients with CD who were non-responsive or intolerant to previous TNF-antagonist therapy. Five randomised controlled trials were included. No statistically significant differences were revealed in clinical response and clinical remission in the induction phase of therapy; neither in clinical remission in a maintenance phase. In addition, primary and secondary non-responder subpopulations showed no significant differences in clinical response. Of note, indirect comparison of ustekinumab and vedolizumab for clinical response in the induction phase of therapy (6 weeks) showed no statistically significant difference specifically in primary anti-TNF non-responders.

In summary, some data, mainly from real-life, indirectly comparing vedolizumab and ustekinumab in CD patients with prior failure to anti-TNF agents, suggest that ustekinumab could be more effective than vedolizumab. However, the following potential limitations (applicable to both individual comparative studies and meta-analyses), should be taken into consideration when interpreting these indirect comparisons:

(a) The number of patients included in each study is relatively small.
(b) The studies are mostly non-randomised, and many of them even retrospective.
(c) The results of the studies are difficult to compare due to differences in patient populations, methodology and statistical analysis. For example, initial trials of infliximab comprised anti-TNF naïve patients, while significant proportions of patients included in trials for recent anti-TNF (or other biologics) had prior anti-TNF exposure.
(d) The results of some studies are difficult to interpret, since only responders to induction therapy were enrolled in the maintenance studies, which were supplemented with responders from open-label cohorts of which no outcomes are presented.
(e) Even though most of the observational studies were adjusted for potential confounding factors performing a propensity score matching, the validity of this analysis relies on the untestable assumption that all confounders were accounted for.
(f) Most of the studies do not include endoscopic follow-up and therefore data on mucosal healing is not available; thus, only clinical effectiveness is generally assessed.
(g) The duration of follow-up is limited; ideally, studies with longer follow-up duration are required to confirm outcomes over a prolonged period.
(h) Finally, most studies indirectly comparing vedolizumab and ustekinumab in anti-TNF experienced patients do not provide effectiveness results according to the type of failure to the anti-TNF treatment (primary failure, loss or response or intolerance) [108].

## 6. Conclusions

**Figure 1:**
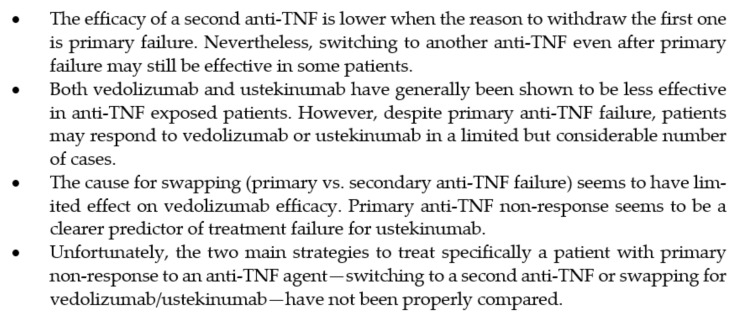
Main conclusions regarding the two main strategies to treat a patient with primary non-response to an anti-TNF agent: switching to a second anti-TNF or swapping for a drug with another mechanisms of action.

Approximately one-third of IBD patients do not respond to anti-TNF therapy, which represents a challenging scenario. In the last few years, novel molecules have become available with mechanisms of action different from anti-TNF blockade, and many more are tested in the therapeutic pipeline. Consequently, therapeutic options for anti-TNF-experienced IBD patients are constantly expanding, creating new opportunities for the management of difficult-to-treat patients. Identifying patients who are most likely to benefit from specific agents is of paramount importance to help best positioning IBD therapies. In the present study, we have reviewed, for the first time, current data on the two main strategies to treat a patient with primary non-response to an anti-TNF agent: switching to a second anti-TNF or swapping for a drug with another mechanisms of action (mainly vedolizumab and ustekinumab). The conclusions of the present review are summarized in Figure 1.

The efficacy of a second anti-TNF seems to depend on the cause for switching, being lower when the reason to withdraw the first anti-TNF is primary failure. Nevertheless, switching to another anti-TNF even after primary failure may still be a valid therapeutic option in some patients, suggesting that primary non-response to anti-TNF therapy may not be necessarily a class-effect phenomenon. In any case, patients who have failed treatment with anti-TNF agents are probably a more refractory group, and this seems to be valid not only for second-line anti-TNF therapy but also for any other rescue biologic agent. Thus, both vedolizumab and ustekinumab have generally been shown to be less effective in anti-TNF exposed patients, although the experience of these biologics in anti-TNF-naïve patients (i.e., in the comparative group) is still quite limited. Nevertheless, despite primary non-response to an anti-TNF drug, patients may respond to vedolizumab or ustekinumab in a limited but considerable number of cases. The cause for swapping (primary vs. secondary anti-TNF failure) seems to have limited effect on the efficacy of vedolizumab treatment, although a lower efficacy of this biologic in patients with primary anti-TNF failure cannot be ruled out. In the case of ustekinumab, anti-TNF primary non-response seems to be a clearer predictor of treatment failure (compared with those patients with loss of response to anti-TNF therapy).

Unfortunately, the two main strategies to treat specifically a patient with primary non-response to an anti-TNF agent—switching to a second anti-TNF or swapping for vedolizumab or ustekinumab—which constitutes the main issue raised in the present article, have not been properly compared (studies are exceptional for vedolizumab and non-existent for ustekinumab). Therefore, no conclusion can be drawn about the best strategy (switch or swap) in the specific scenario of anti-TNF primary non-responders.

Finally, limited data, mainly coming from real-life, indirectly comparing vedolizumab and ustekinumab in CD patients with prior failure to anti-TNF, suggest that ustekinumab could be more effective than vedolizumab. However, these studies (and the meta-analysis including them) suffer from relevant methodological limitations, and do not provide enough information according to the type of failure to the anti-TNF treatment (primary failure, loss or response or intolerance).

In summary, the data reviewed in the present study clearly emphasise the imperative need to carry out head-to-head randomised trials in patients exposed to anti-TNF agents in general, and specifically in those with primary non-response to these agents. These warranted future randomised studies should directly compare the two main strategies to specifically treat a patient with primary non-response to an anti-TNF agent: switching to a second anti-TNF or swapping for a drug with another mechanisms of action. Additionally, these desirable studies should also compare vedolizumab vs. ustekinumab (and vs. tofacitinib) in the setting of anti-TNF primary failure. Only in this way we can obtain reliable information to inform clinicians regarding the optimal positioning of biologic therapies in IBD.

## Figures and Tables

**Table 1:** Studies evaluating the efficacy of a second anti-TNF agent after primary failure with a first one. Efficacy after secondary failure in those studies, if available, is also included.

Author	Year	Disease	1st Anti-TNF (Failed)	2nd Anti-TNF	Remission in Primary Failure	Remission in Secondary Failure
Barthel [20]	2005	CD	IFX	ADA	1/1(100%) at 3 months	NA
Casanova [21]	2020	CD&UC	IFX, ADA, GOL, CER	IFX, ADA, GOL, CER	12/32 (39%) at 14 months	12/28 (42%) at 14 months
Chaparro [22]	2012	CD	ADA	IFX	0/7 (0%)	3/5 (60%)
Clark-Snustad [23]	2019	CD	ADA, GOL	IFX	11/15 (73%) at 3 months	NA
Cordero Ruiz [24]	2011	CD	IFX	ADA	0/2 (0%) at 12 months	14/20 (70%) at 12 months
Cozijnsen [25]	2015	CD (children)	IFX	ADA	1/3 (33%) at 12 months	24/34 (71%) at 12 months
Favale [26]	2019	UC	IFX	ADA	1/3 (33%) at 12 months	8/20 (40%) at 12 months
Fumery [27]	2015	CD	IFX	ADA	1/4 (25%) at 16 months	11/16 (68%) at 16 months
Garcia-Bosch [28]	2017	UC	IFX	ADA	2/6 (33%) at 3 months	25/33 (76%) at 3 months
Ho [29]	2008	CD	IFX	ADA	3/6 (50%) at 12 months	NA
Ho [30]	2009	CD	IFX	ADA	10/29 (33%) at 12 months	NA
Lichtiger [31]	2010	CD	IFX	ADA	4/13 (31%) at 3-9 months	30/75 (40%) at 3-9 months
Lofberg [32]	2012	CD	IFX	ADA	26/89 (29%) at 1 month 33/89 (37%) at 6 months	69/174 (40%) at 6 months
Panaccione [33]	2011	CD	IFX	ADA	1/22 (5%) at 1 month 4/22 (18% at 6 months	NA
R.Grau [34]	2016	CD	IFX, ADA	IFX, ADA	60/118 (51%) at 3 months	NA
Seiderer [35]	2007	CD	IFX	ADA	1/4 (25%) at 2 months	3/4 (75%) at 2 months
Sprakes [36]	2011	CD	IFX	ADA	4/9 (44%) at 6 weeks 4/9 (44%) at 15 months	15/18 (83%) at 6 weeks 12/18 (67%) at 15 months (median)
Swaminath [37]	2009	CD	IFX	ADA	3/6 (50%) at 1.5 months (response, not remission)	NA
Swoger [38]	2010	CD	IFX	ADA	1/9 (11%) at 12 months	NA
Viola [39]	2019	UC	ADA, GOL	IFX	21/60 (35%) at 3 months 20/44 (45%) at 6 months 15/32 (47%) at 12 months	6/14 (43%) at 3 months 8/14 (57%) at 6 months 10/13 (77%) at 12 months

CD: Crohn’s disease; UC: ulcerative colitis; IFX: infliximab; ADA: adalimumab; GOL: golimumab; CER: certolizumab; NA: non-available.

**Table 2:** Efficacy of vedolizumab in primary failures to anti-TNF treatment.

Author	Publication Year	Disease	Response
GEMINI Induction [50,54]	2013	UC	44/82 (54%)
GEMINI maintenance [50,54]	2013	UC	33/83 (40%)
GEMINI 2 and 3 induction [14,53,68]	2013–2019	CD	109/263 (41%)
GEMINI 2 maintenance [14,53]	2013–2019	CD	68/159 (43%)
Favale [26]	2019	CD	6/12 (50%)

Modified from Singh et al. [69]. UC: ulcerative colitis; CD: Crohn’s disease.

**Table 3:** Efficacy of ustekinumab in primary failures to anti-TNF treatment.

Author	Publication Year	Disease	Response
CERTIFI [86]	2012	CD	116/394 (29%)
UNITI 1 [15]	2016	CD	72/249 (29%)

Modified from Singh et al. [69]. UC: ulcerative colitis; CD: Crohn’s disease.

**Table 4:** Meta-analyses comparing the efficacy of different biologic agents for the treatment of inflammatory bowel disease.

Author	Year	Disease	Type of Study	Number of RCTs	Compared Drugs	General Conclusion	Data Specifically in Anti-TNF Failure
Vickers [95]	2016	UC	NMA	8	Anti-TNF, vedolizumab	All biologic treatments were effective during induction. Vedolizumab demonstrated possible clinical benefits in the maintenance setting versus all comparators, irrespective of prior anti-TNF exposure	In anti-TNF-exposed patients, only vedolizumab and adalimumab could be compared. At induction, no significant differences in efficacy were seen. During maintenance, vedolizumab showed higher mucosal healing rates than adalimumab
Cholapranee [96]	2017	CD & UC	MA	12	Anti-TNF, vedolizumab	Anti-TNF and vedolizumab are effective in inducing mucosal healing in UC, with adalimumab being inferior to infliximab. Infliximab and adalimumab were similar in CD	NA
Bonovas [97]	2018	UC	MA	5	Anti-TNF, vedolizumab, tofacitinib	Tofacitinib and biologics are efficacious for UC (only non-experienced anti-TNF patients were included)	NA
Kawalec [98]	2018	CD	MA	5	Vedolizumab, ustekinumab	No significant differences were revealed between vedolizumab and ustekinumab for clinical response and clinical remission in the induction phase, and for remission in the maintenance phase of TNF therapy in refractory patients	No significant differences were revealed in clinical response between primary and secondary non-responders’ subpopulations. In particular, indirect comparison of ustekinumab and vedolizumab for clinical response in the induction phase of therapy (6 weeks) specifically in primary non-responders was non-statistically significant
Pagnini [99]	2018	CD	NMA	8	Anti-TNF, vedolizumab, ustekinumab	In anti-TNF-experienced CD patients, secondary biological administration may be efficient	In anti-TNF-experienced CD patients, secondary biological administration may be efficient, although no specific agent seems to outperform the others. However, primary non-responders treated with anti-TNF after failure were not included
Trigo-Vicente [100]	2018	UC	NMA	14	Anti-TNF, vedolizumab, tofacitinib	infliximab may be the best therapeutic option for moderate-to-severe UC. Vedolizumab appears superior to golimumab and adalimumab	NA
Singh [101]	2018	CD	NMA	8	Anti-TNF, vedolizumab, ustekinumab	Infliximab or adalimumab may be preferred first-line agents, and ustekinumab a preferred second-line agent, for induction of remission in patients with moderate-severe CD	In patients with prior anti-TNF exposure, adalimumab (low quality of evidence, in patients with prior response or intolerance to anti-TNF agents) and ustekinumab were ranked highest for induction of clinical remission
Singh [69]	2018	CD & UC	MA	8	Anti-TNF, vedolizumab, ustekinumab	Patients with primary non-response to anti-TNF agents are less likely to respond to second-line non-TNF biologics, as compared with patients who discontinued therapy due to secondary loss of response or intolerance	As compared with patients who discontinued prior anti-TNF due to intolerance, patients with prior primary non-response were 24% less likely to achieve remission with second-line biologics. As compared with patients who discontinued prior anti-TNF due to loss of response, patients with prior primary non-response were 27% less likely to achieve remission with induction therapy with second-line biologics, particularly with ustekinumab. There was no difference in response to vedolizumab in patients with prior primary non-response or loss of response to anti-TNF agents
Singht [102]	2018	UC	NMA	16	Anti-TNF, vedolizumab, tofacitinib	Infliximab and vedolizumab are ranked highest as first-line agents	Tofacitinib is ranked highest as second-line agent, for induction of remission in patients with moderate-severe UC
Varu [103]	2019	CD	NMA	13	Anti-TNF, ustekinumab	Ustekinumab was associated with the highest likelihood of reaching response or remission at 1 year compared with adalimumab and vedolizumab	NA
Lohan [104]	2019	UC	NMA	17	Anti-TNF, vedolizumab, tofacitinib	Tofacitinib is an efficacious treatment for moderately to severely active UC (in TNF-naïve patients, all therapies were more efficacious than placebo)	In TNF-exposed patients, only tofacitinib was significantly more efficacious than placebo as induction therapy, and only tofacitinib and vedolizumab were significantly more efficacious than placebo as maintenance therapies
Welty [105]	2020	UC	NMA	21	Anti-TNF, vedolizumab, ustekinumab, tofacitinib	Higher likelihood of response, remission and endoscopic-mucosal healing at 1 year with ustekinumab versus comparators in the non-biologic failure population	In biologic-experienced patients (4 RCTs), ustekinumab was the most effective treatment
Zhou [106]	2020	UC	NMA	16	Anti-TNF, vedolizumab, tofacitinib	Of the biological agents, vedolizumab and infliximab were the most effective	NA
Singh [107]	2020	UC	NMA	22	Anti-TNF, vedolizumab, ustekinumab, tofacitinib	Infliximab was ranked highest in biologic-naïve patients	In patients with prior exposure to TNF antagonists, ustekinumab and tofacitinib were ranked highest for induction of clinical remission and were superior to vedolizumab and adalimumab in patients with moderate to severe UC

CD: Crohn’s disease; UC: ulcerative colitis; MA: meta-analysis; NMA: network meta-analysis; RCTs: randomised controlled trials; NA: non-available.

## Supplementary Materials

The following are available online at https://www.mdpi.com/article/10.3390/jcm10225318/s1, Table S1: Criteria used for primary failure by the studies (included in Table 1) which reported their results stratified by type of failure (primary vs. secondary).

## Abbreviations

anti-TNF	Anti-tumour necrosis factor
CD	Crohn’s disease
IBD	Inflammatory bowel disease
iv	Intravenous
sc	Subcutaneous
UC	Ulcerative colitis
