# Supplementary material for: Primary Failure to an Anti-TNF Agent in Inflammatory Bowel Disease: Switch (to a Second Anti-TNF Agent) or Swap (for Another Mechanism of Action)?
Source: J Clin Med. 2021 Nov 15;10(22):5318. doi: 10.3390/jcm10225318 (PMC8625924; doi:10.3390/jcm10225318)
Supplement: Supplementary file 1 [file jcm-10-05318-s001.zip › jcm-1453642-supplementary.pdf]

Table S1. Criteria used for primary failure by the studies (included in Table 1) which reported their results stratified by type of failure (primary vs. secondary).

Author	Criteria for primary failure
Casanova [21]	It was considered that the patient had a primary failure if she/he did not achieve remission after having received the induction doses of the anti-TNF (non-responders and partial responders)
Chaparro [22]	For luminal disease, response to adalimumab and infliximab was evaluated using the Harvey-Bradshaw index (HBI) four weeks after the first dose. Partial response was defined as a decrease in the HBI of more than 3 points. Remission was defined as a HBI below or equal to 4 without steroids. In perianal Crohn's disease, complete response was defined as closure of all fistulas and partial response as a 50% or more reduction in the number of draining fistulas.
Cordero Ruiz [24]	No specific definition was provided (just stated "lack of initial response to infliximab")
Cozijnsen [25]	No specific definition was provided (just stated "non-response to infliximab")
Favale [26]	Primary failure was defined as the lack of clinical response based on physician's global assessment (PGA) within 10 weeks from the first induction dose.
Fumery [27]	Primary failure was defined as the absence of clinical remission or clinical response at 6 months after adalimumab initiation
Garcia-Bosch [28]	No specific definition was provided (just stated "primary non-responders to infliximab")
Lichtiger [31]	Primary non-responders to infliximab were defined as patients who never responded to their initial exposure to infliximab
Lofberg [32]	Classification of study subjects as primary non-responders to infliximab was determined by the investigator
Seiderer [35]	No specific definition was provided (just stated "infliximab-refractory")
Sprakes [36]	Primary non-response was defined as individuals who did not respond to the standard three dose induction with infliximab
Viola [39]	No specific definition was provided (just stated "patients unresponsive to adalimumab or golimumab who were switched to infliximab")
